# Depression and HIV in Botswana: A Population-Based Study on Gender-Specific Socioeconomic and Behavioral Correlates

**DOI:** 10.1371/journal.pone.0014252

**Published:** 2010-12-08

**Authors:** Reshma Gupta, Madhavi Dandu, Laura Packel, George Rutherford, Karen Leiter, Nthabiseng Phaladze, Fiona Percy-de Korte, Vincent Iacopino, Sheri D. Weiser

**Affiliations:** 1 Department of Medicine, University of California San Francisco, San Francisco, California, United States of America; 2 Division of Hospital Medicine, Department of Medicine, University of California San Francisco, San Francisco, California, United States of America; 3 Global Health Sciences, University of California San Francisco, San Francisco, California, United States of America; 4 Department of Epidemiology and Biostatistics, University of California San Francisco, San Francisco, California, United States of America; 5 Physicians for Human Rights, Cambridge, Massachusetts, United States of America; 6 School of Nursing, University of Botswana, Gaborone, Botswana; 7 Department of Medicine, University of Minnesota, Minneapolis, Minnesota, United States of America; 8 Division of HIV/AIDS and Positive Health Program, Department of Medicine, University of California San Francisco, San Francisco, California, United States of America; Instituto de Pesquisa Clinica Evandro Chagas, FIOCRUZ, Brazil

## Abstract

**Background:**

Depression is a leading contributor to the burden of disease worldwide, a critical barrier to HIV prevention and a common serious HIV co-morbidity. However, depression screening and treatment are limited in sub-Saharan Africa, and there are few population-level studies examining the prevalence and gender-specific factors associated with depression.

**Methods:**

We conducted a cross-sectional population-based study of 18–49 year-old adults from five districts in Botswana with the highest prevalence of HIV-infection. We examined the prevalence of depressive symptoms, using a Hopkins Symptom Checklist for Depression (HSCL-D) score of ≥1.75 to define depression, and correlates of depression using multivariate logistic regression stratified by sex.

**Results:**

Of 1,268 participants surveyed, 25.3% of women and 31.4% of men had depression. Among women, lower education (adjusted odds ratio [AOR] 2.07, 95% confidence interval [1.30–3.32]), higher income (1.77 [1.09–2.86]), and lack of control in sexual decision-making (2.35 [1.46–3.81]) were positively associated with depression. Among men, being single (1.95 [1.02–3.74]), living in a rural area (1.63 [1.02–2.65]), having frequent visits to a health provider (3.29 [1.88–5.74]), anticipated HIV stigma (fearing discrimination if HIV status was revealed) (2.04 [1.27–3.29]), and intergenerational sex (2.28 [1.17–4.41]) were independently associated with depression.

**Discussion:**

Depression is highly prevalent in Botswana, and its correlates are gender-specific. Our findings suggest multiple targets for screening and prevention of depression and highlight the need to integrate mental health counseling and treatment into primary health care to decrease morbidity and improve HIV management efforts.

## Introduction

Depression is a significant contributor to the burden of disease in developing countries. In 2004, the World Health Organization had listed depression as the third leading contributor to the burden of disease worldwide and the number one contributor to the burden of disease among women. It is projected to become the number one burden of disease worldwide by 2030. The prevalence of depression is higher in developing countries [1], [2] compared to developed countries [3].

Depression often leads to worse health outcomes. It has been correlated with increased healthcare utilization in high resource countries [4] and lower productivity, poor relationships, and poor quality of life in both low [5] and high resource countries [6]. Depression and stress are also associated with increased risk of heart disease and increased mortality [7], [8].

Depression is a critical barrier that must be addressed when discussing HIV prevention as the two diseases are intrinsically linked and exacerbate each other. Depression is associated with inconsistent condom use that may contribute to increased HIV transmission in both low resource and high resource settings [9], [10], [11]. Having a diagnosis of HIV has been found to worsen depression in North America and Europe [12], [13]. In turn, depression worsens HIV-related health outcomes. Depression has been associated with steeper declines in CD4 counts [14], [15], greater risk of developing HIV-dementia [16], worse antiretroviral medication adherence [17], and more rapid progression to AIDS and death [14], [16], [18]. Lack of awareness surrounding depression prevents affected individuals from receiving proper treatment and services due to limited and misallocated treatment resources [19].

Botswana provides a unique opportunity to study depression in a country with a high prevalence of HIV. In 2006, it had the second highest prevalence of HIV worldwide with 24% of adults infected between the ages of 15–49 [20]. In this region, women are disproportionately affected with women 15 years and older having approximately 2 times the prevalence and young women (15–24 years old) having more than 3 times the prevalence of HIV-infection as men [21]. HIV-stigma is common with 38% of the adult population reporting at least one stigmatizing attitude [22], and there are high rates of high-risk sexual behavior with 38% reporting to have had unprotected sex over the past year [23]. Botswana has been a leader in sub-Saharan Africa launching the first national antiretroviral treatment program in 2002; however, initial efforts to reduce HIV transmission have had limited success [24].

There are few data from nationally-representative population-based studies examining correlates of depression or whether HIV risk behaviors are associated with depression in sub-Saharan Africa. The limited number of existing studies addressing correlates of depression have been mostly small, focused on sub-populations, and have not examined many correlates that are particularly relevant in countries with high HIV prevalence [25], [26], [27], [28]. There are also no previous studies on depression in Botswana. We therefore studied the prevalence and correlates of depression in a population-based sample in Botswana to help inform programs targeting depression to improve health outcomes, quality of life, and reduce HIV transmission risk.

## Methods

In November and December 2004, we conducted a cross-sectional population-based study using a structured 234-item survey among a probably sample of 18–49 year-old adults selected from the five districts of Botswana with the highest number of HIV-infected individuals (Gaborone, Kweneng East, Francistown, Serowe/Palapye, and Tutume). These 5 districts comprised 43% of Botswana's population of 1.7 million (725,000 individuals). We used a stratified 2-stage probability sample design for the selection of households with the assistance of the Central Statistics Office at the Ministry of Finance and Development Planning in Botswana as described elsewhere [23]. We calculated a target sample of 1,200 households and added an additional 15% over-sampling for an anticipated 85% response rate. At the pre-selected households, we used random number tables to select one male or female adult who met inclusion criteria. Up to two repeat visits were made, and no replacements were made if participants could not be contacted after two visits.

### Study Procedures

Participants were included in the study if they were adults aged 18 through 49 years, were legal residents of Botswana from one of the five districts with the highest number of HIV-infected individuals, were English or Setswana speaking, had no cognitive disabilities that may have interfered with their understanding of the informed consent procedure, and had adequate privacy to conduct the interviews.

Surveys and consent forms were translated into English and then back-translated into Setswana to ensure accuracy. We obtained written consent from all study participants. Interviews were conducted in either English or Setswana in a private setting and lasted 45–60 minutes each. Following the recommendations of local collaborators, we did not ask about HIV status in order to maximize response rates and minimize participant discomfort. The Committee on Human Research at the University of California, San Francisco, and the Botswana Ministry of Health Research and Development Committee approved all study procedures.

### Measures

In our surveys, we asked about socio-demographics, health status and health care utilization, HIV knowledge, experiences with HIV testing, HIV-related stigma, depression, gender-discriminatory beliefs, and HIV risk behaviors. Our primary outcome, depression, was measured using the 15-item Hopkins Symptom Checklist for Depression (HSCL-D) [29], which has been validated previously in a number of international settings in Africa [30]. Respondents were asked whether they were bothered by each symptom on a 4-point scale from “not at all” (1) to “extremely.” A score of 1.75 or above corresponds to a diagnosis of depression according to this measure. The internal consistency of this measure was high in this sample, with a Cronbach alpha of 0.85.

We measured HIV-related stigma using two measures of stigma that have been used successfully in studies among individuals of unknown HIV serostatus: endorsing stigmatizing attitudes towards people living with HIV/AIDS (PLWHA) and anticipated HIV stigma, defined as fearing discrimination if HIV status was revealed. Both of these measures have been described in depth elsewhere [22]. Briefly, participants were asked seven questions on stigmatizing attitudes towards PLWHA that were adapted from the UNAIDS General Population Survey and the Department of Health Services AIDS module. Individuals were considered to have stigmatizing attitudes if they endorsed one or more items on this scale. Since individuals may not always openly discuss stigmatizing views toward PLWHA due to social desirability bias, we added a measure of “anticipated HIV stigma,” based on a 9-item index. We scored participants as having anticipated HIV stigma if they believed that one or more negative social experiences would occur if they were to test positive for HIV and divulge their status to others. This index had high internal reliability with a Cronbach alpha of 0.74.

HIV risk behaviors were all measured over the 12 months prior to the interview. Inconsistent condom use was defined as having not used a condom at any time in the previous 12 months. Sex exchange was defined amongst women as having received money, food, or other resources in exchange for sex; it was defined among men as having given money, food, or other resources in exchange for sex or having had sex with a commercial sex worker. Intergenerational relationships were defined as having a sexual partner whose age was at least 10 years younger among male participants or 10 years older among female participants. Lack of control in sexual decision-making was defined as having partner(s) who usually or always make the decision as to whether or not to have sex.

### Statistical Analysis

We used descriptive statistics to outline study respondent characteristics, prevalence of depression, and mean HSCL-D scores. Categorical variables were compared with Chi^2^ tests and continuous variables by t-tests. We used bivariate and multivariate logistic regression to examine independent correlates of depression. All analyses were stratified by sex since prior studies have shown that correlates of depression are gender-specific [28].

Our decisions about which risk factors to investigate were guided by an ecological multiple risk/protective factor model that has been proposed for conceptualizing risk factors related to depressive symptoms [31]. Our central thematic premise relies on depression being a result of mutual interactions among an individual (socio-demographics), his or her immediate environment and health (health and healthcare status), and larger social and cultural contexts in which he or she is embedded (attitudes and practices). We also considered previously identified risk factors from other developing countries for inclusion into our model and examined correlates from 3 categories [4], [9], [25], [28], [32], [33], [34], [35], [36], [37], [38]: *Socio-*d*emographic correlates*: (1) age (continuous), (2) sex (male or female), (3) monthly household income (<1000 pula or >1000 pula), (4) education (≥high school or < high school), (5) occupation (employed or unemployed), (6) residence type (rural, urban, or urban village), (7) marital status (married, living with partner, or other), (8) food insufficiency defined as having had problems obtaining enough food to eat over the past 12 months (dichotomous), a question modified from a previously validated measure of food insufficiency, which also used a single question [39]; *Health status and health utilization correlates:* (9) self-reported health status (poor/fair, good, very good/excellent), (10) frequency of visit to a modern healthcare provider over the previous 12 months (0, 1–2, >3); *Correlates related to attitudes and practices:* (11) endorsing stigmatizing attitudes (dichotomous), (12) anticipating HIV stigma (dichotomous), (13) alcohol use (no/moderate drinking defined as having had 1–7 drinks/week for women and 1–14 drinks/week for men or problem/heavy drinking defined as 8–14 drinks/k week for women and 15–21 drinks/week for men), (14) inconsistent condom use (dichotomous); (15) sex exchange (dichotomous); (16) intergenerational relationships (dichotomous); (17) lack of control in sexual decision-making (dichotomous). As a result of the dearth of literature related to depression in high prevalence HIV settings in sub-Saharan Africa, our analyses are intended to be exploratory and hypothesis generating for future studies.

All variables with p-values < = 0.25 in bivariate analysis were included in our multivariate model. as recommended by Hosmer and Lemeshow [40]. We conducted an additional analysis to compare the participant characteristics used in our analysis (listed in Table 1) with individuals excluded from our analysis due to incomplete depression screens. Data were analyzed with Stata statistical software, version 10.0 (Stata Corporation LP, College Station, TX). Regression diagnostic procedures yielded no evidence of multi-collinearity or overly influential outliers in any of the models. No individual survey question used for this analysis had more than 3% missing data.

**Table 1:** Characteristics of Participants by Gender (N = 1178).

		N = 1178	Male N = 580	Female N = 597	
*Characteristic*	*Subcategory*	*All N (%) or* mean (+/− SD)	N (%) or mean (+/−SD)	N (%) or mean (+/−SD)	p-value
**Socio-demographic Characteristics**					
Age, yr		31.6+/−11.2	31.5+/−11.2	31.8+/−11.2	p = 0.63
Sex		—	580 (49.3)	597 (50.7)	—
Income	<1000 Pula	530 (45.2)	229 (39.5)	301 (50.9)	p<0.01
	> = 1000 Pula	642 (54.8)	351 (60.5)	290 (49.1)	
Education	Less than high school	531 (45.5)	253 (44.1)	277 (46.7)	p = 0.34
	High school or more	637 (54.5)	321 (55.9)	316 (53.3)	
Occupation	Unemployed	355 (30.5)	156 (27.1)	199 (33.8)	p = 0.01
	Employed	810 (69.5)	419 (72.9)	390 (66.2)	
Locale of Residence	Urban	507 (43.0)	228 (39.3)	279 (46.7)	p = .0.40
	Urban village	331 (28.1)	173 (29.8)	157 (26.3)	
	Rural	340 (28.9)	179 (30.9)	161 (27.0)	
Marital Status	Married	231 (19.6)	100 (17.3)	130 (21.8)	p = 0.01
	Not married living with partner	314 (26.7)	141 (24.4)	173 (29.0)	
	Not married not living with partner	631 (53.7)	338 (58.4)	293 (49.2)	
Food insufficiency		273 (23.2)	103 (17.8)	170 (28.5)	p<0.01
**Depression**	Overall no. with depression	334 (28.4)	182 (31.4)	151 (25.3)	p = 0.02
	Mean HSCL-D score^a^	—	1.6+/−0.5	1.6+/−0.5	P = 0.34
**Health Status and Healthcare Utilization**					
Self-reported Health Status	Excellent/Very Good/Good	830 (70.8)	397 (68.9)	432 (72.6)	p = 0.17
	Fair/Poor	342 (29.1)	179 (31.1)	163 (27.4)	
Freq. visit to modern healthcare provider:	0	260 (22.1)	152 (26.2)	108 (18.2)	p<0.01
	1–2	521 (44.3)	266 (45.9)	255 (42.9)	
	>3	394 (33.5)	162 (27.9)	231 (38.9)	
**Attitudes and Practices**					
Endorsing stigmatizing attitudes		447 (38.0)	227 (39.1)	220 (37.0)	p = 0.45
Anticipated HIV stigma		821 (71.0)	423 (73.7)	398 (68.4)	p = 0.05
Alcohol abuse	No/moderate drinking	785 (67.50)	343 (59.97)	442 (74.79)	p<0.01
	Problem/heavy drinking	378 (32.50)	229 (40.03)	149 (25.02)	
Inconsistent condom use over past 12 months		438 (38.4)	211 (37.6)	227 (39.1)	p = 0.60
Sex exchange		122 (10.5)	77 (13.5)	45 (7.7)	p<0.01
Intergenerational relationships		164 (14.1)	54 (9.5)	110 (18.6)	p<0.01
Lack of control in sexual decision-making		157 (13.6)	9 (1.6)	148 (25.2)	p<0.01

a HSCL-D stands for Hopkins Symptom Checklist for Depression (15-item scale).

## Results

**Figure 1:**
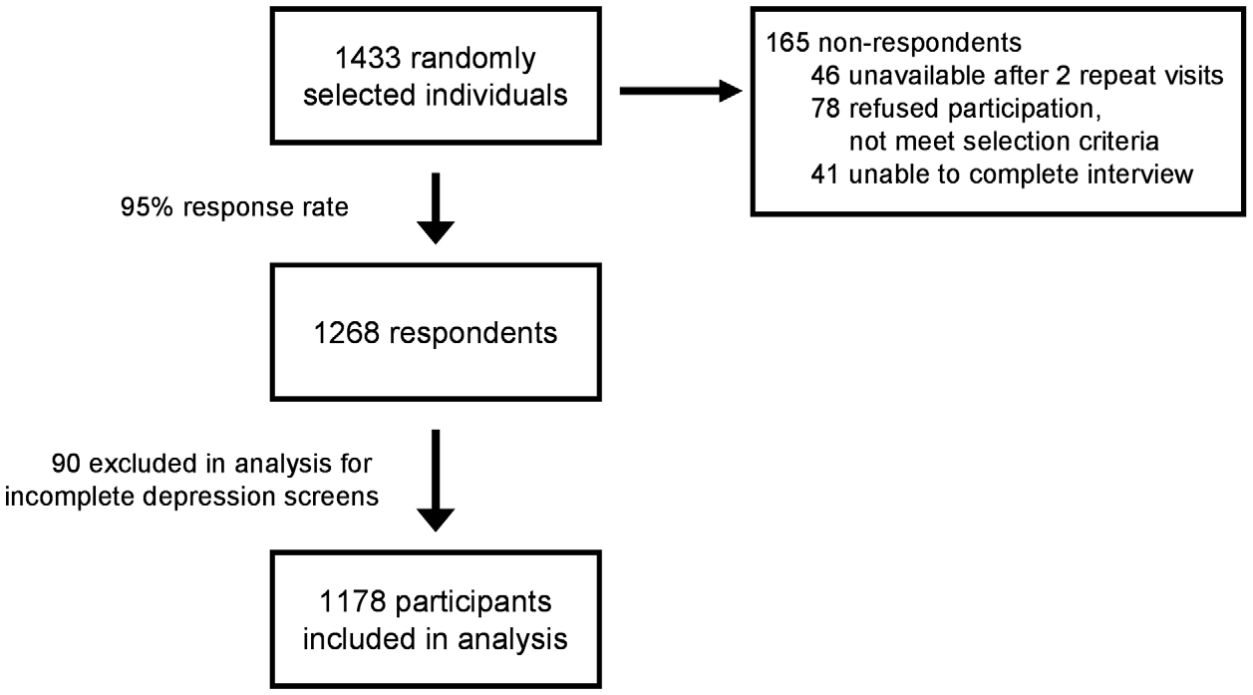
Participant Inclusion Flowchart.

Of 1,433 randomly selected individuals in Botswana, 1,268 (89%) completed the survey. Among the 165 non-respondents, 46 (28%) were unavailable after two repeat visits, 78 (47%) refused to participate or did not meet selection criteria, and 41 (25%) were unable to complete the interview. Of the 1,268 respondents, 90 (7%) were excluded due to having incomplete depression screens, leaving 1168 study participants for this analysis (Figure 1). The 90 individuals who were excluded from our analysis differed significantly in sex (female>men) compared to the included participants (p<0.02) but were otherwise similar demographically.

Demographic and behavioral characteristics of the study participants, stratified by gender, are shown in Table 1. The average age of participants was 32 years old and 597 (50.7%) were women. Women had lower incomes (50.9% compared to 39.5% of men reporting incomes of <1000 P/month), higher unemployment (33.8% compared to 27.1% among men), and greater food insufficiency (28.5% compared to 17.8% among men). Women had more frequent health provider visits than men (38.9% with 3 or more visits compared to 27.9% among men). Both women and men reported high rates of fair/poor health status (27.4% of women and 31.1% of men). Both women and men had high rates of endorsing stigmatizing attitudes (37.0% of women and 39.1% of men) and anticipated HIV stigma (68.4% of women and 73.7% of men). Men had higher rates of both types of HIV stigma when looking at individual components of stigma variables as well as composite variables. There was a high prevalence of problem/heavy drinking, especially among men (40.0% compared to 25.0% among women). Additionally, we found a high prevalence of HIV transmission risk behaviors. Women had a higher prevalence of intergenerational sexual relationships (18.6% compared to 9.5% among men) and lack of control in sexual decision-making (25.2% compared to 1.6% among men). Similar to other studies in the region, we also found high rates of inconsistent condom use in the overall population (38.4%), but no significant differences between sexes (Table 1).

Three hundred thirty-four (28%) respondents had HSCL-D scores consistent with depression including 151 (25%) women and 182 (31%) men (p<0.02). Both men and women had mean HSCL-D scores of 1.6 (Table 1). Frequencies of HSCL-D components are listed in Table 2. Among women, in unadjusted analysis, lower educational status, unemployment, food insufficiency, fair/poor health status, and lack on control in sexual decision-making were associated with a higher odds of screening positive for depression (Table 3). In adjusted analysis, women with lower educational status had a two times higher odds of depression (AOR = 2.07, 95% CI = 1.30–3.32), and those with a higher income had a 77% higher odds of having depression (AOR = 1.77, 95% CI = 1.09–2.86) (Table 4). Lack control in their sexual relationships was the strongest correlate, with more than a two times higher odds of depression (AOR = 2.35, 95% CI 1.46–3.81).

**Table 2:** 15-item Hopkins Symptom Checklist for Depression [HSCL-D] Participant Prevalence.

	Male (N = 580)	Female (N = 597)
HSCL-D Items:	n (%)^a^	n (%)^a^
Feeling low in energy, slowed down	274 (47.2)	277 (46.4)
Blaming yourself for things	278 (47.9)	257 (43.1)
Crying easily	74 (12.8)	149 (25.0)
Loss of sexual interest or pleasure	259 (44.7)	254 (42.6)
Poor appetite	293 (50.5)	318 (53.3)
Difficulty falling asleep, staying asleep	308 (53.1)	311 (52.1)
Feeling hopeless about the future	206 (35.5)	182 (30.5)
Feeling down or blue	280 (48.3)	264 (44.2)
Feeling lonely	288 (49.7)	292 (48.9)
Thoughts of ending your life	11 (1.9)	21 (3.5)
Feelings of being trapped or caught	190 (32.8)	126 (21.1)
Worry too much about things	315 (54.3)	326 (54.6)
Feeling no interest in things	251 (43.3)	211 (35.3)
Feeling everything is an effort	250 (43.1)	206 (34.5)
Feeling worthless	167 (28.8)	128 (21.4)

a Frequencies show participants answering if they have been bothered by symptoms either “a little,” “quite a bit,” or “extremely.”

**Table 3:** Unadjusted Factors Associated with Depression among Adults in Botswana Stratified by Gender.

*Characteristic*		Depression	
		Males (N = 580)	Females (N = 597)
	*Subcategory*	OR (95% CI)	OR (95% CI)
**Socio-demographic Characteristics**			
Age, yr		1.0 (0.98–1.01)- p = 0.42	1.0 (0.98–1.02)- p = 0.93
Male Sex		1.4 (1.1–1.7)- p = 0.02*	Referent group
Income	<1000 Pula	Referent group	Referent group
	> = 1000 Pula	1.3 (0.9–1.9)- p = 0.15	1.0 (0.7–1.4)- p = 0.98
Education	Less than high school	1.2 (0.9–1.8)- p = 0.23	2.3 (1.5–3.3)- <0.01**
	High school or more	Referent group	Referent group
Occupation	Unemployed	1.3 (0.9–1.9)- p = 0.21	1.6 (1.1–2.3)- p = 0.02*
	Employed	Referent group	Referent group
Locale of Residence	Urban	Referent group	Referent group
	Urban village	1.2 (0.8–1.8)- p = 0.49	1.0 (0.6–1.5)- p = 0.91
	Rural	1.4 (0.9–2.2)- p = 0.10	0.7 (0.4–1.1)- p = 0.11
Marital Status	Married	Referent group	Referent group
	Not married living with partner	1.7 (0.9–3.0)- p = 0.08	2.1 (1.2–3.5)- p = 0.01*
	Not married not living with partner	1.7 (1.0–2.8)- p = 0.06	1.2 (0.7–1.9)- p = 0.56
Food Insufficiency		0.8 (0.5–1.3)- p = 0.45	1.8 (1.2–2.6) p<0.01**
**Health Status and Healthcare Utilization**			
Self-reported Health Status	Excellent/Very Good/Good	Referent group	Referent group
	Fair/Poor	1.4 (0.9–2.0)- p = 0.12	1.6 (1.1–2.4)- p = 0.03*
Freq. visit to modern healthcare provider:	0	Referent group	Referent group
	1–2	1.8 (1.1–2.9)- p = 0.02*	1.4 (0.8–2.5)- p = 0.22
	>3	3.2 (1.9–5.3)- p<0.01**	1.6 (0.9–2.8)- p = 0.09
**Attitudes and Practices**			
Endorsing stigmatizing attitudes		0.9 (0.6–1.2)- p = 0.44	1.0 (0.7–1.5)- p = 0.97
Anticipated HIV stigma		1.9 (1.2–2.9)- p = 0.01**	1.2 (0.8–1.8)- p = 0.42
Alcohol abuse	No/moderate drinking	Referent group	Referent group
	Problem/heavy drinking	1.5 (1.0–2.1) p = 0.04*	0.8 (0.5–1.2) p = 0.29
Inconsistent condom use over past 12 months		1.6 (1.1–2.3) p = 0.01*	1.2 (0.8–1.8)- p = 0.30
Sex exchange		1.8 (1.1–2.9) p = 0.03*	1.1 (0.5–2.2)- p = 0.82
Intergenerational relationships		2.9 (1.6–5.1) p<0.01**	1.3 (0.8–2.1)- p = 0.22
Lack of control in sexual decision-making		0.3 (0.03–2.2)- p = 0.22	2.8 (1.9–4.1)- p<0.01**

*p-value<0.05,

**p-value<0.01, p-value<0.25.

**Table 4:** Adjusted Factors Associated with Depression among Adults in Botswana Stratified by Gender.

*Characteristic*		Depression	
		Males (N = 558)	Females (N = 580)
	*Subcategory*	AOR (95% CI)	AOR (95% CI)
**Socio-demographic Characteristics**			
Age, yr		0.99 (0.97–1.01) p = 0.45	1.00 (0.98–1.01) p = 0.62
Male Sex		—	—
Income	<1000 Pula	Referent group	Referent group
	> = 1000 Pula	1.37 (0.86–2.19) p = 0.18	1.77 (1.09–2.86) p = 0.02*
Education	Less than high school	1.38 (0.90–2.12) p = 0.14	2.07 (1.30–3.32) p = 0.002***
	High school or more	Referent group	Referent group
Occupation	Unemployed	1.34 (0.84–2.15) p = 0.21	1.33 (0.85–2.09) p = 0.21
	Employed	Referent group	Referent group
Locale of Residence	Urban	Referent group	Referent group
	Urban village	1.18 (0.73–1.91) p = 0.49	0.80 (0.48–1.35) p = 0.41
	Rural	1.63 (1.02–2.65) p = 0.04*	0.65 (0.38–1.12) p = 0.12
Marital Status	Married	Referent group	Referent group
	Not married living with partner	1.71 (0.89–3.29) p = 0.11	1.66 (0.89–3.11) p = 0.11
	Not married not living with partner	1.95(1.02–3.74) p = 0.04*	1.13 (0.61–2.09) p = 0.70
Food insufficiency		0.75 (0.43–1.33) p = 0.33	1.62 (1.00–2.62) p = 0.05
**Health Status and Healthcare Utilization**			
Self-reported Health Status	Excellent/Very Good/Good	Referent group	Referent group
	Fair/Poor	1.43 (0.94–2.21) p = 0.10	1.24 (0.78–1.98) p = 0.36
Freq. visit to modern healthcare provider:	0	Referent group	Referent group
	1–2	1.69 (1.01–2.81) p = 0.04*	1.30 (0.70–2.40) p = 0.41
	>3	3.29 (1.88–5.74) p<0.001***	1.23 (0.66–2.30) p = 0.51
**Attitudes and Practices**			
Anticipated HIV stigma		2.04 (1.27–3.29) p = 0.003**	1.14 (0.71–1.81) p = 0.59
Alcohol abuse	No/moderate drinking	Referent group	Referent group
	Problem/heavy drinking	1.02 (0.66–1.55) p = 0.94	0.79 (0.45–1.36) p = 0.39
Inconsistent condom use over past 12 months		1.23 (0.78–1.95) p = 0.37	0.82 (0.52–1.31) p = 0.41
Sex exchange		1.18 (0.66–2.12) p = 0.57	0.89 (0.38–2.09) p = 0.79
Intergenerational relationships		2.28 (1.17–4.41) p = 0.02*	1.13 (0.65–1.98) p = 0.66
Lack of control in sexual decision-making		0.18 (0.01–2.50) p = 0.20	2.35 (1.46–3.81) p<0.001***

*p-value<0.05,

**p-value<0.01,

***p-value<0.0025.

Among men, in unadjusted analysis, having greater than two or three healthcare visits in the last year, anticipated HIV stigma, inconsistent condom use, sex exchange, and having intergenerational relationships were associated with higher odds of depression (Table 3). In adjusted analysis, coming from a rural area was correlated with 63% higher odds of having depression (95% CI = 1.02–2.65), and being unmarried and not living with a partner was correlated with approximately two times higher odds of screening positive for depression (AOR = 1.95, 95% CI = 1.02–3.74) (Table 4). Having more than three visits to a modern health provider was the strongest correlate, with more than three times higher odds of depression (AOR = 3.29, 95% CI = 1.88–5.74). Additionally, men who anticipated HIV stigma and those who reported intergenerational sex each had more than two times higher odds of depression (AOR = 2.04, 95% CI 1.27–3.29, and AOR = 2.28, 95% CI 1.17–4.41, respectively).

## Discussion

To our knowledge, this is one of the first population-based studies to examine the prevalence of depression and to systematically explore gender-specific correlates of depression in a high prevalence HIV setting in sub-Saharan Africa. We found that over 30% of men and 25% of women in the five regions surveyed in Botswana screened positive for depression, which is between two and four times the prevalence found in the United States [3], [41]. These findings are consistent with several other smaller studies in the region where HIV prevalence is also high; 21% of individuals in a small study in Uganda and 31% of individuals from a small study in Zimbabwe screened positive for depression [30], [42]. A population-based study in South Africa and another in Zambia found prevalence of depression between 5–15% [25], [43]. These results underscore our need to better screen for and treat depression within Botswana and in other settings with a high prevalence of HIV. Our finding that men had a higher prevalence of depression compared to women differs from prior studies in the region that had found either no differences in the prevalence of depression by gender or a higher prevalence among women [28], [34]. Potential explanations for this finding are that compared to women in this region, men often have less social support [34] and access healthcare less frequently [44], which have been shown to be correlated with depression in prior literature from low resource settings including among PLWHA [34].

Earlier studies on correlates of depression in sub-Saharan Africa have focused primarily on socio-demographic factors. Our findings on socio-demographic correlates were similar to those found in literature from both resource-poor and resource-rich settings. We found that lower educational status among women and being unmarried among men were associated with depression, similar to previous studies [25], [33], [35], [36], [37]. Additionally, we saw a trend towards an association between depression and both unemployment [28], [35], [37] and food insufficiency [38] as previously reported in studies from resource poor and rich countries. We found that women with higher incomes had a higher prevalence of depression. This finding contrasts with some previous studies that have reported the converse [28], [35], [36] but corresponds to findings among specific US subpopulations (e.g. obese women) [45]. Men from rural areas in Botswana had greater odds of depression, consistent with a prior study from Ethiopia [32].

Our study highlights associations between depression and increased healthcare utilization and worse clinical outcomes, which can be useful to guide future studies in the region. Similar to prior studies, we found that depression was associated with increased frequency of visits to health providers among men [4], suggesting the need to screen for and treat depression among frequent users of health care services. We also saw a trend towards an association between depression and having fair/poor self-reported health status among women, which has been predictive of developing chronic disease and mortality in other studies [46]. It is possible that depression is a marker for having chronic medical conditions, which in turn can lead to increased health utilization. Further studies are needed to explore the direction of this association and address potential mediators.

Depression has been identified as a major contributor to sexual risk behavior and HIV infection in studies from the US [11] and a few smaller studies in South Africa [9], [10]. Our study is one of the first to find an association between depression and various risky sexual practices in a nationally representative sample in sub-Saharan Africa. We tested four components of risky sexual behavior and found that, among women, lack of control in sexual decision-making correlated with depression in adjusted analysis. Previous research from this region has demonstrated that women experience power inequities, lack of negotiating power and compromised agency within sexual relationships. This finding is consistent with previous research conducted in South Africa that found that having joint-decision making power in relationships is associated with lower odds of depression [36]. In a culture where sexual violence is pervasive with six of 10 women being lifetime survivors of domestic violence, this may reflect social constructs of gender that legitimize potential victimization of women leading to feelings of lower self-worth among women and a higher prevalence of depression. In addition to leading directly to depression, it is also plausible that lack of sexual control can indirectly lead to depression via engaging in risky sex if people do not feel good about their choices and practices. A final possibility is that women who are depressed feel less empowered to assert control over their sexual relationships. Due to the cross-sectional nature of this study, were unable to fully tease apart the complexity of the relationships between lack of control in sexual decision-making and depression. Future studies should use validated measures of sexual relationship control and longitudinal study designs to determine the direction of these associations, and the factors that may mediate these relationships.

Among men, we found that having intergenerational sex correlated with depression in adjusted analyses. Having intergenerational relationships may be a marker of inequitable gender beliefs and practices [47], and intergenerational sex in turn has been associated with sexual violence and sexual risk-taking behavior in many studies [47], [48], [49], [50]. Since intergenerational relationships have been reported to increase male self-esteem and social standing [51], it is possible that depression may make men more likely to seek out intergenerational sexual relationships. Our findings highlight the need to target depression as part of HIV prevention efforts and also suggest that addressing gender power imbalances may decrease depression among both men and women.

Our study is one of the first to demonstrate a strong association between anticipated HIV stigma (the respondent's expectation that he or she would be stigmatized for having HIV) and depression in a resource-poor setting. We found this association among men and a trend towards an association with depression among women. Several small studies from sub-Saharan Africa have reported correlations between depression and enacted stigma (experiencing discrimination) or internalized stigma (attributes discredited by society become internalized and accepted as valid by PLWHA) [34], measures that are typically used in studies where HIV status of participants are known. Our findings are unique since we measure HIV stigma at a nationally representative population level in Africa where the burden of HIV stigma is so great [52], and also because we are the first to report an association between anticipated HIV stigma and depression in the general population. In the country with the second to highest HIV prevalence in the world, it is not difficult to imagine why anticipating negative responses to HIV could contribute to symptoms of depression among the general population. Both depression and HIV stigma have in turn been associated with poor physical and mental health outcomes and increased risk of HIV transmission [52]. Therefore, the association between anticipated HIV stigma and depression further strengthens the rationale for targeting HIV stigma as part of comprehensive HIV prevention and care programs.

There were a few important limitations to this study. Since the design of the study was cross-sectional, we cannot draw conclusions about the directionality of the associations between the variables. This study was intended to be hypothesis generating to guide future studies. Since we did not interview individuals from more rural districts of Botswana and since Botswana has a relatively high per capita income and comparatively extensive healthcare infrastructure, we can not necessarily generalize these results to neighboring countries. We used self-reported responses, which may lead to social desirability bias. However under-reporting of some risky sexual behaviors and other practices would bias results toward the null hypothesis, so true associations may be stronger than our data indicate. Additionally, participant HIV status, a health factor that may affect mental health, health practices and economic status, was not asked directly in our interviews. Our measure of depression is based on a screening tool for depression and not a diagnostic tool. Finally, we do not account for severity of depression since the HSCL-D tool does not allow for clinically meaningful separations in depression severity. The direction of effect between depression and risky sexual practices may depend on the severity and type of depression. For example, a prior study showed that major depression was correlated with less sexual activity, while dysthymic disorder was associated with increased unprotected sex [53]. Further research will be needed to evaluate variation in correlates of depression based on depression severity. Additionally, variables including lack of control in sexual decision-making, intergenerational sex, and HIV-related stigma have complex social constructs and will require additional study to fully understand their associations with depression.

In summary, we found that depression is highly prevalent in Botswana, and its correlates are gender-specific. Our findings suggest multiple targets for screening for and preventing depression and highlight the need to integrate mental health counseling, screening, and treatment interventions into primary health care. Treatment options for depression are currently limited in Botswana with few primary care providers trained to detect psychiatric disorders. Nonetheless, depression is a treatable condition and interventions using either antidepressants or psychotherapy can be successful and inexpensive in resources-low settings [19], [54]. Treatment of depression will likely lead to less transmission of HIV and improved outcomes for those already infected.

## References

[1] Thavichachart N, Intoh P, Thavichachart T, Meksupa O, Tangwongchai S (2001). Epidemiological survey of mental disorders and knowledge attitude practice upon mental health among people in Bangkok Metropolis.. J Med Assoc Thai.

[2] Garcia-Alvarez R (1986). Epidemiology of depression in Latin America.. Psychopathology.

[3] Weissman MM, Bland RC, Canino GJ, Faravelli C, Greenwald S (1996). Cross-national epidemiology of major depression and bipolar disorder.. JAMA.

[4] Rowan PJ, Davidson K, Campbell JA, Dobrez DG, MacLean DR (2002). Depressive symptoms predict medical care utilization in a population-based sample.. Psychol Med.

[5] Gureje O, Kola L, Afolabi E (2007). Epidemiology of major depressive disorder in elderly Nigerians in the Ibadan Study of Ageing: a community-based survey.. Lancet.

[6] Hughes J, Jelsma J, Maclean E, Darder M, Tinise X (2004). The health-related quality of life of people living with HIV/AIDS.. Disabil Rehabil.

[7] Antelman G, Kaaya S, Wei R, Mbwambo J, Msamanga GI (2007). Depressive symptoms increase risk of HIV disease progression and mortality among women in Tanzania.. J Acquir Immune Defic Syndr.

[8] Rosengren A, Hawken S, Ounpuu S, Sliwa K, Zubaid M (2004). Association of psychosocial risk factors with risk of acute myocardial infarction in 11119 cases and 13648 controls from 52 countries (the INTERHEART study): case-control study.. Lancet.

[9] Smit J, Myer L, Middelkoop K, Seedat S, Wood R (2006). Mental health and sexual risk behaviours in a South African township: a community-based cross-sectional study.. Public Health.

[10] Olley BO, Seedat S, Stein DJ (2006). Persistence of psychiatric disorders in a cohort of HIV/AIDS patients in South Africa: a 6-month follow-up study.. J Psychosom Res.

[11] Meade CS, Sikkema KJ (2005). HIV risk behavior among adults with severe mental illness: a systematic review.. Clin Psychol Rev.

[12] Boarts JM, Buckley-Fischer BA, Armelie AP, Bogart LM, Delahanty DL (2009). The impact of HIV diagnosis-related vs. non-diagnosis related trauma on PTSD, depression, medication adherence, and HIV disease markers.. J Evid Based Soc Work.

[13] Hand GA, Phillips KD, Dudgeon WD (2006). Perceived stress in HIV-infected individuals: physiological and psychological correlates.. AIDS Care.

[14] Ickovics JR, Hamburger ME, Vlahov D, Schoenbaum EE, Schuman P (2001). Mortality, CD4 cell count decline, and depressive symptoms among HIV-seropositive women: longitudinal analysis from the HIV Epidemiology Research Study.. JAMA.

[15] Burack JH, Barrett DC, Stall RD, Chesney MA, Ekstrand ML (1993). Depressive symptoms and CD4 lymphocyte decline among HIV-infected men.. JAMA.

[16] Farinpour R, Miller EN, Satz P, Selnes OA, Cohen BA (2003). Psychosocial risk factors of HIV morbidity and mortality: findings from the Multicenter AIDS Cohort Study (MACS).. J Clin Exp Neuropsychol.

[17] Holzemer WL, Corless IB, Nokes KM, Turner JG, Brown MA (1999). Predictors of self-reported adherence in persons living with HIV disease.. AIDS Patient Care STDS.

[18] Evans DL, Leserman J, Perkins DO, Stern RA, Murphy C (1997). Severe life stress as a predictor of early disease progression in HIV infection.. Am J Psychiatry.

[19] Demyttenaere K, Bruffaerts R, Posada-Villa J, Gasquet I, Kovess V (2004). Prevalence, severity, and unmet need for treatment of mental disorders in the World Health Organization World Mental Health Surveys.. JAMA.

[20] National ARV project team MoH, Botswana (2006). The Masa Antiretroviral Therapy Program in Botswana: Patient Enrollment Update..

[21] UNAIDS (2008). Epidemiological Fact Sheet on HIV and AIDS:.

[22] Wolfe WR, Weiser SD, Leiter K, Steward WT, Percy-de Korte F (2008). The impact of universal access to antiretroviral therapy on HIV stigma in Botswana.. Am J Public Health.

[23] Weiser SD, Leiter K, Heisler M, McFarland W, Percy-de Korte F (2006). A population-based study on alcohol and high-risk sexual behaviors in Botswana.. PLoS Med.

[24] Smart T (2005). Former manager of Botswana's antiretroviral treatment programme describes barriers to rapid scale-up and suggest possible solutions at 2nd Annual African AIDS Conference..

[25] Chipimo PJ, Fylkesnes K (2009). Mental distress in the general population in Zambia: impact of HIV and social factors.. BMC Public Health.

[26] Cooper PJ, Tomlinson M, Swartz L, Woolgar M, Murray L (1999). Post-partum depression and the mother-infant relationship in a South African peri-urban settlement.. Br J Psychiatry.

[27] Uwakwe R (2000). The pattern of psychiatric disorders among the aged in a selected community in Nigeria.. Int J Geriatr Psychiatry.

[28] Patel V, Todd C, Winston M, Gwanzura F, Simunyu E (1997). Common mental disorders in primary care in Harare, Zimbabwe: associations and risk factors.. Br J Psychiatry.

[29] Derogatis LR, Lipman RS, Rickels K, Uhlenhuth EH, Covi L (1974). The Hopkins Symptom Checklist (HSCL). A measure of primary symptom dimensions.. Mod Probl Pharmacopsychiatry.

[30] Bolton P, Wilk CM, Ndogoni L (2004). Assessment of depression prevalence in rural Uganda using symptom and function criteria.. Soc Psychiatry Psychiatr Epidemiol.

[31] Bronfenbrenner U (1979). The ecology of human development..

[32] Deyessa N, Berhane Y, Alem A, Hogberg U, Kullgren G (2008). Depression among women in rural Ethiopia as related to socioeconomic factors: a community-based study on women in reproductive age groups.. Scand J Public Health.

[33] Fleischer NL, Fernald LC, Hubbard AE (2007). Depressive symptoms in low-income women in rural Mexico.. Epidemiology.

[34] Simbayi LC, Kalichman S, Strebel A, Cloete A, Henda N (2007). Internalized stigma, discrimination, and depression among men and women living with HIV/AIDS in Cape Town, South Africa.. Soc Sci Med.

[35] Myer L, Stein DJ, Grimsrud A, Seedat S, Williams DR (2008). Social determinants of psychological distress in a nationally-representative sample of South African adults.. Social Science & Medicine.

[36] Hamad R, Fernald LC, Karlan DS, Zinman J (2008). Social and economic correlates of depressive symptoms and perceived stress in South African adults.. J Epidemiol Community Health.

[37] Costello EJ (1991). Married with children: predictors of mental and physical health in middle-aged women.. Psychiatry.

[38] Boris NW, Brown LA, Thurman TR, Rice JC, Snider LM (2008). Depressive symptoms in youth heads of household in Rwanda: correlates and implications for intervention.. Arch Pediatr Adolesc Med.

[39] Alaimo K, Olson CM, Frongillo EA (2002). Family food insufficiency, but not low family income, is positively associated with dysthymia and suicide symptoms in adolescents.. J Nutr.

[40] Hosmer DW, Lemeshow S (1989). Applied Logistic Regression..

[41] Kessler RC, Chiu WT, Demler O, Merikangas KR, Walters EE (2005). Prevalence, severity, and comorbidity of 12-month DSM-IV disorders in the National Comorbidity Survey Replication.. Arch Gen Psychiatry.

[42] Abas MA, Broadhead JC (1997). Depression and anxiety among women in an urban setting in Zimbabwe.. Psychol Med.

[43] Williams DR, Herman A, Stein DJ, Heeringa SG, Jackson PB (2008). Twelve-month mental disorders in South Africa: prevalence, service use and demographic correlates in the population-based South African Stress and Health Study.. Psychol Med.

[44] Bankole A, Singh S, Hussain R, Wulf D (2004). The sexual, marital and fathering behavior of men in sub-Saharan Africa..

[45] Stunkard AJ, Faith MS, Allison KC (2003). Depression and obesity.. Biol Psychiatry.

[46] Mossey JM, Shapiro E (1982). Self-rated health: a predictor of mortality among the elderly.. Am J Public Health.

[47] Leclerc-Madlala S (2008). Age-disparate and intergenerational sex in southern Africa: the dynamics of hypervulnerability.. AIDS.

[48] Glynn JR, Carael M, Auvert B, Kahindo M, Chege J (2001). Why do young women have a much higher prevalence of HIV than young men? A study in Kisumu, Kenya and Ndola, Zambia.. AIDS.

[49] Longfield K, Glick A, Waithaka M, Berman J (2004). Relationships between older men and younger women: implications for STIs/HIV in Kenya.. Stud Fam Plann.

[50] Hallman K (2004). Socioeconomic disadvantage and unsafe sexual behaviors among young women and men in South Africa..

[51] Brown J, Sorrell J, Raffaelli M (2005). An exploratory study of constructions of masculinity, sexuality and HIV/AIDS in Namibia, Southern Africa.. Cult Health Sex.

[52] Holzemer WL, Uys L, Makoae L, Stewart A, Phetlhu R (2007). A conceptual model of HIV/AIDS stigma from five African countries.. J Adv Nurs.

[53] Rogers G, Curry M, Oddy J, Pratt N, Beilby J (2003). Depressive disorders and unprotected casual anal sex among Australian homosexually active men in primary care.. HIV Med.

[54] Jamison DT, Feachem RG, Makgoba MW, Bos ER, Baingana FK (2006). Disease and mortality in Sub-Saharan Africa. 2nd Edition ed..

